# Prediction of gait intention from pre-movement EEG signals: a feasibility study

**DOI:** 10.1186/s12984-020-00675-5

**Published:** 2020-04-16

**Authors:** S. M. Shafiul Hasan, Masudur R. Siddiquee, Roozbeh Atri, Rodrigo Ramon, J. Sebastian Marquez, Ou Bai

**Affiliations:** Department of Electrical and Computer Engineering, Florida International University, Miami, Florida USA

**Keywords:** Electroencephalography (EEG), Brain-computer interface (BCI), Gait intention prediction, Discrete wavelet transform, Hjorth parameters

## Abstract

**Background:**

Prediction of Gait intention from pre-movement Electroencephalography (EEG) signals is a vital step in developing a real-time Brain-computer Interface (BCI) for a proper neuro-rehabilitation system. In that respect, this paper investigates the feasibility of a fully predictive methodology to detect the intention to start and stop a gait cycle by utilizing EEG signals obtained before the event occurrence.

**Methods:**

An eight-channel, custom-made, EEG system with electrodes placed around the sensorimotor cortex was used to acquire EEG data from six healthy subjects and two amputees. A discrete wavelet transform-based method was employed to capture event related information in alpha and beta bands in the time-frequency domain. The Hjorth parameters, namely activity, mobility, and complexity, were extracted as features while a two-sample unpaired Wilcoxon test was used to get rid of redundant features for better classification accuracy. The feature set thus obtained was then used to classify between ’walk vs. stop’ and ’rest vs. start’ classes using support vector machine (SVM) classifier with RBF kernel in a ten-fold cross-validation scheme.

**Results:**

Using a fully predictive intention detection system, 76.41±4.47*%* accuracy, 72.85±7.48*%* sensitivity, and 79.93±5.50*%* specificity were achieved for ’rest vs. start’ classification. While for ’walk vs. stop’ classification, the obtained mean accuracy, sensitivity, and specificity were 74.12±4.12*%*, 70.24±6.45*%*, and 77.78±7.01*%* respectively. Overall average True Positive Rate achieved by this methodology was 72.06±8.27*%* with 1.45 False Positives/min.

**Conclusion:**

Extensive simulations and resulting classification results show that it is possible to achieve statistically similar intention detection accuracy using either only pre-movement EEG features or trans-movement EEG features. The classifier performance shows the potential of the proposed methodology to predict human movement intention exclusively from the pre-movement EEG signal to be applied in real-life prosthetic and neuro-rehabilitation systems.

## Background

Locomotion impairments resulting from cerebrovascular accidents or stroke and brain trauma create a severe handicap for many people, especially the elderlies [1]. Besides, lower limb amputation due to trauma or diabetes results in reduced mobility, thus severely affecting people’s quality of life. Various assistive orthosis and prosthesis devices have been developed in recent years to help people with diminished motor abilities [2–7]. Although several Electromyography (EMG)-based studies have been carried out [8, 9], Brain-Computer Interface (BCI) or Brain-Machine Interfaces (BMI) have been more extensively investigated in the recent years in the scope of gait rehabilitation due to their great prospect in understanding and analyzing gait-related brain rhythms and Event-Related Potentials (ERPs). As Electroencephalography (EEG) signals can act as a real-time projection of brain’s motor activity during gait, EEG- based gait studies hold significant potential in achieving early prediction of future movement plans which researchers can readily utilize for more effective rehabilitation of motor-impaired persons providing them with necessary motor capabilities.

Prediction of human movement intention is highly significant for successful gait rehabilitation. In a BCI-based rehabilitation system, the brain waves are extracted, processed, and translated to control an assistive device. For an effective assistive system, it is critical to detect the movement intention as early as possible to provide the system with enough time to adapt to the requirement of the individual [10]. There have been two majorly reported neural features related to movement intention detection. Those are Movement-Related Cortical Potential (MRCP) [11–13] and Event-Related Synchronization/ Desynchronization (ERS/ ERD) [14, 15]. MRCP corresponding to self-paced movement is known as Bereitschafts Potential (BP), and it is characterized by a slow decrease in EEG amplitude over the primary motor cortex within at least 0.5 s preceding the movement initiation. On the other hand, ERD is defined as a decrease in spectral power 0.5- 2 s before movement onset reported most in the mu (8-12 Hz) and beta (13-30 Hz) frequency bands of the brain wave [16–18]. The limits of the frequency bands may differ across different authors. These features have been used as physiological triggers to activate and operate various assistive devices [19–21].

Although ERD [22, 23] and MRCP [24–26] based studies have been carried out quite extensively in understanding unique aspects of motor cortex activation, both of these modalities have some drawbacks. MRCP provides timing information about different stages of movement planning and execution. But it is a very subtle change in near DC frequencies and it takes multiple repetitions of the same trial to extract useful and reliable gait-related information from MRCP [27]. On the other hand, ERD has been shown to be detectable from single trial EEG, but the main disadvantage of using ERD as a control signal for assistive devices is that it does not provide precise timing information about different stages of movement planning, preparation, and execution. Moreover, ERD/ERS requires a reliable steady state baseline to correctly detect power changes correlated to movement intention. The current studies [28, 29] either use EEG signal from both before and after the movement initiation and termination or use a large portion of pre-movement EEG data for proper baseline information. Such systems are, therefore, impractical in online real-time BCI application and may become prone to erroneous or delayed detection of intention in the presence of sudden intention to move. To address the shortcoming of the traditional features, in this paper Hjorth parameters, namely activity, mobility and complexity are proposed as features to obtain instantaneous time-frequency information related to gait intention in a window by window approach without the necessity of baseline selection.

Besides, very few studies have explored pre-movement state for healthy subjects in the context of upper or lower limb function [28, 30–35]. The pre movement neurological changes in the EEG signal have been identified and studied previously. However, the possibility of predicting human voluntary gait intention in a real-time BCI scenario is yet to be extensively investigated. Also, understanding the exclusive pre-movement EEG signal parameters for healthy subjects as well as amputees is yet to be explored. Moreover, detecting gait intention before the movement onset or termination would be a very critical feature for online BCI systems for rehabilitation. Currently, early and accurate detection of self- paced movement intention for real-time BCI application remains a daunting challenge. Early and accurate detection of gait intention would give the BCI system necessary time to validate the authenticity of the predicted intention and adapt the parameters of corresponding assistive devices thus ensuring safe and natural gait rehabilitation. That is why a fully predictive BCI would always be preferred over a BCI which would only detect gait intention after the gait had already happened. As such this study would explore the feasibility of predicting gait intention from pre movement EEG data only. The data analysis and classification were done in an offline scenario to investigate whether it is possible to separate EEG data corresponding to gait ’start’ or ’stop’ intention from EEG data related to steady state ’walking’ or ’resting’ respectively. Moreover, a trans-movement EEG data structure was also evaluated for the same classification task to analyze whether use of only pre movement EEG data caused any statistical decline in system performance. This study would work as a good starting point for future online implementation of BCI for gait rehabilitation.

In this paper, a Wavelet Transform based intention detection methodology was proposed to address the challenges discussed in the above paragraphs by utilizing Hjorth parameters as features. Several time windows before movement initiation and termination were used for classification, which is more similar to real life situations where the intention to move or stop can be abrupt. Moreover, a brief duration of data after the movement initiation or termination were also included with the pre-movement data windows in a separate classification scheme to examine the change in detection performance and to validate the feasibility of the proposed fully predictive system. Wavelet Transform is chosen due to its ability to provide better time-frequency resolution than conventional signal processing tools like Fast Fourier Transform (FFT). The Hjorth parameter is a convenient tool to extract useful information in the time-frequency domain and also has the advantage of minimal computational complexity compared to other time-frequency analysis tools, e.g., Short Time Fourier Transform (STFT) [36]. These parameters have been used in several EEG– based studies across various applications, such as upper body movement intention detection [36] Alzheimer’s study [37], seizure lateralization [38], emotion recognition [39], mental task classification [40]. However, the Hjorth parameters have not been utilized in the scope of self-paced movement intention detection to the knowledge of the authors. From that background, the performance of Hjorth parameters was investigated in detecting self-paced lower limb movement intention. The hypothesis of this study is: It is possible to predict intention of voluntary gait initiation and termination by using pre-movement EEG signals only.

## Methods

### Experimental procedure

Seven healthy individuals and two amputees (Seven male and two female, mean age = 32.6 years and SD = 10.41 years) participated in the experiment. None of the participants had any known history of neurological disorder. The experimental protocol was approved by the Institutional Review Board (IRB) of Florida International University, and Hunter Holmes McGuire VA Medical Center. Also, signed consent papers were obtained from subjects. The demographic and physiological information of the subjects is summarized in Table 1.

**Table 1:** Demographic characteristics of the subjects

Subjects	Gender	Age	Weight (kg)	Height (cm)	Amputated limb
S1	Male	26	96	171	N/A
S2	Female	23	62	158	N/A
S3	Male	29	71	170	N/A
S4	Male	33	62	152	N/A
S5	Male	30	98	185	N/A
S6	Male	26	61	170	N/A
S7	Female	26	61	165	N/A
A1	Male	47	92	170	Right foot; Trans-Tibial
A2	Male	53	115	167	Right foot; Trans-Tibial

The experiments on healthy subjects were carried out in the Human Cyber-Physical Systems (HCPS) Laboratory at Florida International University. While, the amputee subjects carried out the experiment in Hunter Holmes McGuire VA Medical Center, Richmond, Virginia. The experimental procedure was designed to detect the intention of movement starting and stopping. For this purpose, all the participants were asked to execute several starts and stops of gait while walking on level grounds in a self-paced manner. There were no audio or visual cues offered to the participants because the presence of audio or visual cues might corrupt the gait-related EEG signals. The subjects were allowed to start and stop walking according to their will. However, it was made sure that the duration of walking and resting periods were at least 5 s. This duration was set to ensure the extraction of uncorrupted and distinctive features. To ensure the minimum duration of walking and resting, the subjects were instructed and trained about the duration in a separate short session before the beginning of the actual experiment. In this session, the subjects were asked to complete 5 cycles of gait and then stop walking. While in the rest state, they were instructed to count from 1 to 10 before starting to walk again. In the introductory session, when the subjects could walk and rest for the minimal amount of time according to the instruction they were given, they were then allowed to start the experimental session.

In the experimental session, each of the subjects carried out approximately 140 repetitions of gait cycles in as many runs as they needed. The number of necessary runs varied across the participants. In each run, the following tasks were repeated periodically: Rest, Start walking, Stop, and Rest again. The subjects were allowed to take a rest after every run for as long as they needed. Moreover, as the amputated subjects would find it challenging to complete too many starting and stopping cycles due to probable fatigue, discomfort and more extended inter-session rest periods, they were asked to continue the experiment for as long as they feel comfortable to carry on. That is why the number of gait initiation and terminations trials for the amputated subjects were less than that for the healthy subjects.

### Data acquisition

**Fig. 1:**
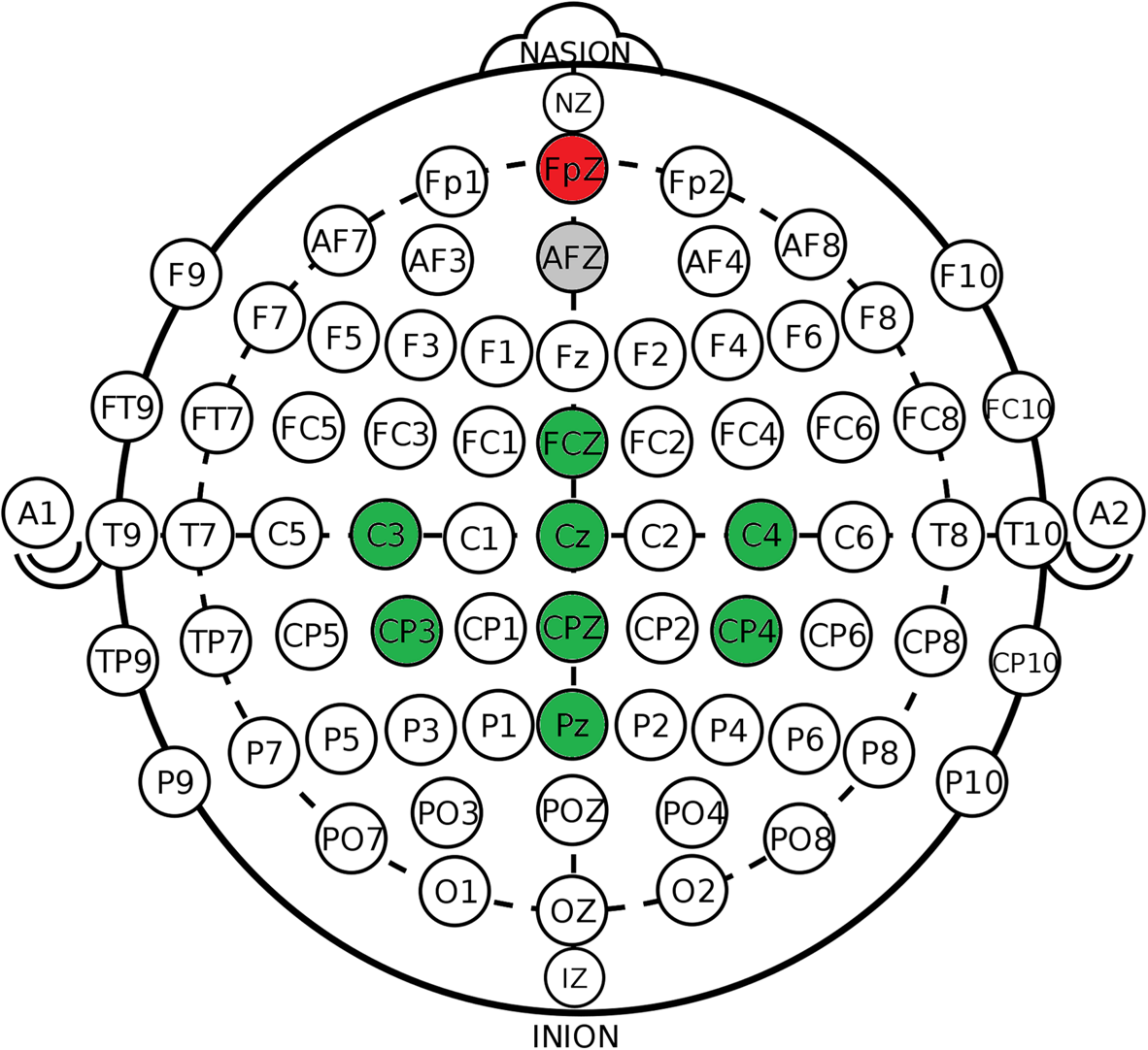
The eight channel electrode system in the International 10-20 system. The green marked channels are the data acquisition channels while the grey marked channel AFz is the ground channel, and the red marked channel FpZ is the reference channel. The image is adapted from the article in [39]

An active electrode system (actiCAP developed by Brainproducts GmbH) was used to collect eight-channel EEG data from all the subjects. A custom made data acquisition board utilizing ADS1299 (Texas Instruments) amplifier was used to amplify the EEG data. The electrodes were placed at Cz, C3, C4, CP3, CP4, FCz, CPz, and Pz, according to the International 10-20 system. The reference and ground electrodes were placed at FPz and AFz, respectively. Figure 1 shows the placement of the electrodes. Before data acquisition, a proper amount of conductive gel was applied to ensure that the impedances of all the electrodes were below a manufacturer-recommended value. The sampling frequency of the EEG data acquisition system was set at 500 Hz.

In addition to that, one channel of Electromyography (EMG) was also recorded using an ADS1292 amplifier at 1000 samples per second. The EMG channel was placed at the mid-belly of right leg Tibialis Anterior (TA) muscle with the reference electrode placed on the bony surface of the right knee. TA was chosen because it is one of the muscles which activates the earliest during a gait cycle [41]. The EMG signal was bandpass pass filtered with cut off frequencies at 20 and 400 Hz using a 4th order Butterworth filter.

A custom made pair of in-sole pressure sensors which was used in a previous study reported in [42] were placed inside the shoes of the subjects to capture which phase of gait they were in at any particular moment. All the boards were also equipped with built-in nine-axis Inertial Measurement Unit (IMU) sensors (MPU-9250, InvenSense) for detecting motion artifacts so that adaptive filtering could be used [43, 44]. The sensors included an accelerometer, a gyroscope, and a magnetometer. The timing information of starting and stopping of gait was acquired from the in-sole pressure sensors, and the corresponding EMG of Tibialis Anterior used to verify the time information acquired from the pressure sensor.

### Preprocessing

As EEG signal is highly prone to noise and also is non- stationary, proper preprocessing tools are needed to extract valuable event related information. In this work, a well-known MATLAB-based EEG processing toolbox EEGLAB [45] was utilized to preprocess the acquired EEG data. For increasing computational efficiency, the signal was downsampled to 250 Hz. Then, the data were high pass filtered by a primary FIR filter with 1 Hz cutoff frequency to get rid of the DC drift in the data. The signal was cleared off line noise at 60 Hz by using a notch filter. The cleansing process of the EEG signal was carried out in two steps using the EEGLAB toolbox: Artifact Subspace Reconstruction (ASR) [46] and Independent Component Analysis (ICA). The ASR algorithm is a non-stationary method which uses sliding window PCA to remove unusual large-amplitude noise or artifacts. The usage of ASR increases data stationarity and makes the data suitable for ICA operation.

In this paper, ASR was used for two purposes: bad channel rejection and removal of short-time high-amplitude artifacts in continuous data. A channel was rejected if (1) it had a flat signal for more than 5 s or (2) was poorly correlated with adjacent channels. The threshold of the cross-correlation was set at 0.7 for all the subjects. To estimate the signal of one channel from contaminating signals of adjacent channels, the SD value for repair bursts using ASR was set to 10. The value was chosen in such as a way that it was ’small enough to remove activities from artifacts and eye-related components and large enough to retain signals from brain-related components’ according to the study in [47].

**Fig. 2:**
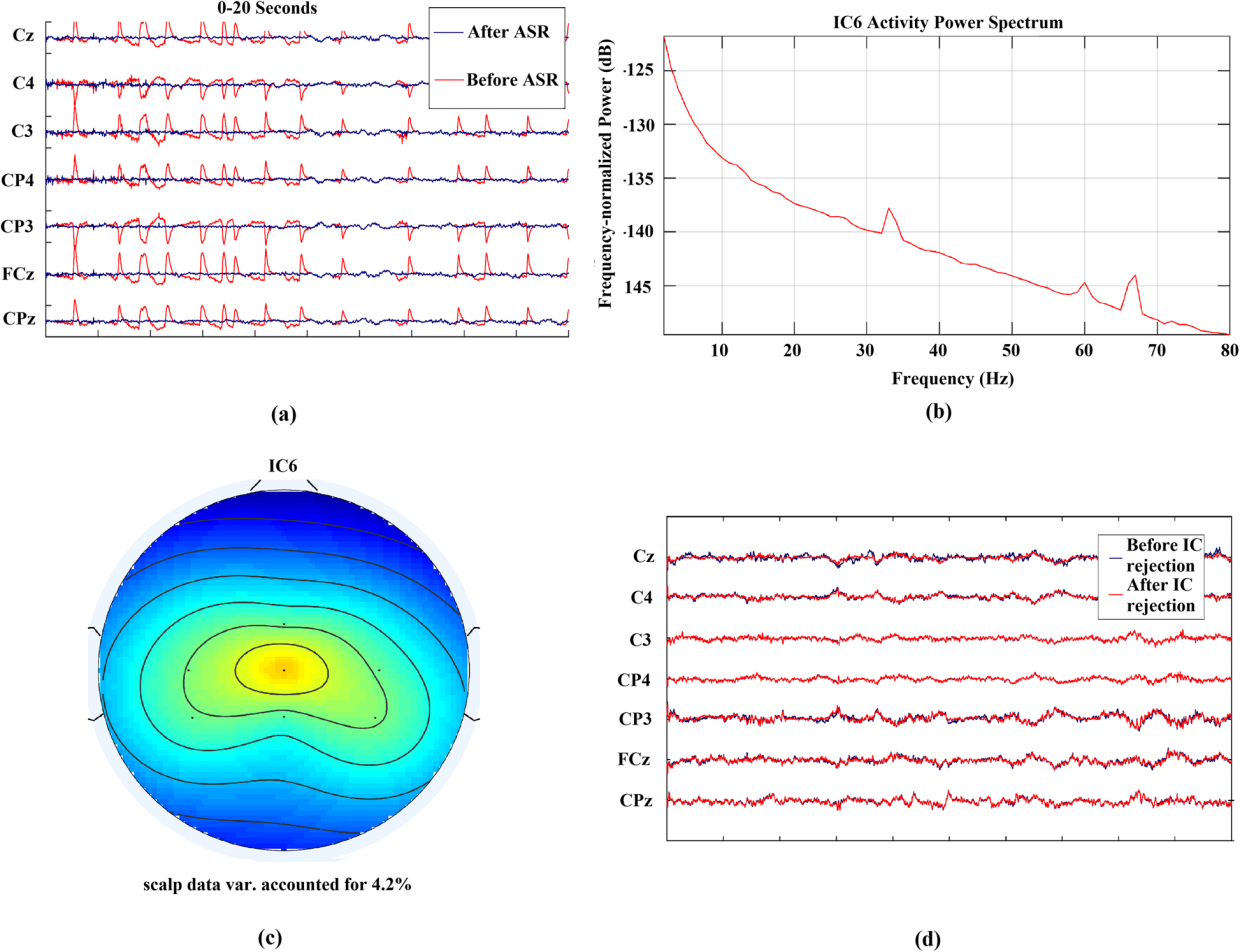
The cleaning process of EEG data from subject 5. **a** Shows the performance of ASR in removing eye movement artifacts and unnatural high amplitude noise. Moreover, channel Pz was marked as a flat channel and was rejected by the algorithm. **b** and **c** shows the power spectrum and scalp distribution of a rejected Independent Component. The IC was rejected due to its low contribution to the scalp data variance and unusual peaks at higher frequencies like 60 and 70 Hz. **d** shows the EEG signal obtained after ASR operation and artifactual IC rejection

After the ASR operation, the processed EEG signal was re-referenced to a common average as a part of the preprocessing process. The re-referenced EEG signal was then ready for ICA operation. A study in [48] reviews some of the msot used Independent Component Analysis method for artifact removal from EEG signals. In the proposed methodology, a variant of ICA called the Adaptive Mixture ICA knows as AMICA was used for processing. AMICA is a binary program for performing ICA decomposition on the input signal with multiple ICA models [49]. AMICA achieves better ICA decomposition than other ICA approaches as reported in [50]. Moreover, multi-model AMICA can be used as a data-driven approach to address the non-stationarity and dynamic changes of continuous EEG data [51]. The resulting independent components thus obtained were then inspected, and artefactual IC’s were rejected by visual inspection. The artifacts might include muscle or heart components, channel noise, line noise, or others. After the rejection of artifacts, the cleaned data were used for further processing and classification scheme. Figure 2 shows different stages of cleaning of the artifact-laden EEG data.

### Discrete wavelet transform

The EEG signal cleaned by ASR and ICA was then analyzed by discrete wavelet transform to look into the signal properties in the time-frequency domain. A 5-level decomposition of the EEG data was carried out using the Daubechies 4 or ’db4’ as the mother wavelet, and the corresponding frequency ranges are shown in Table 2. The detail coefficients of the third and fourth level of decomposition correspond to the beta and alpha band of brain waves, respectively. As these two brain waves are the most informative about human gait stages as reported in multiple studies, the second and third level detail coefficients were reconstructed to get the beta and alpha band EEG signals. The resulting alpha and beta band signals were used for further processing and feature extraction.

**Table 2:** Different levels of coefficients and their corresponding brain waves

Coefficients	Frequency Range (approx.)	Sub-band
First level detail coefficient (cD1)	62.5-125	-
Second level detail coefficient (cD2)	31.25-62.5	Gamma
Third level detail coefficient (cD3)	15.63-31.25	Beta
Fourth level detail coefficient (cD4)	7.81-15.63	Alpha
Fourth level approximate coefficients (cA4)	0-7.81	Delta and theta

### Data segmentation

**Fig. 3:**
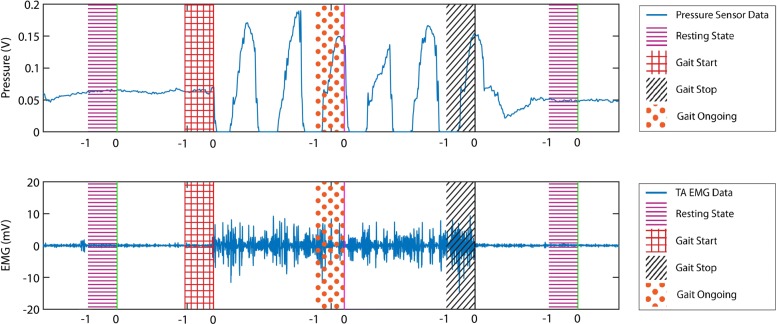
A 20-s segment of pressure sensor and right TA EMG data from Subject 1. The red line in the figure corresponds to the time of gait starting, while the black line corresponds to the time of gait stopping. The resting and walking times were chosen by taking equidistant points from two adjacent gait starting and stopping times. After marking all crucial time points, data windows of different lengths, and relative positions corresponding to those events were taken for further processing and feature extraction. In the figure, the segmentation procedure of [-1,0] data interval is shown. The data windows were marked by -1 and 0 where 0 denotes the extracted event times and -1 denotes the time points one second before the event. The extracted data windows were labeled to the corresponding events. After that, two two-class classification problems were addressed: ’Rest vs. Start’ and ’Walk vs. Stop.’ This segmentation and windowing approach ensured no overlapping between data windows corresponding to different classes reducing the chance of data contamination

The overall data segmentation procedure is summarized in Fig. 3. The red and black lines line in the figure corresponds of gait starting and stopping respectively. Equidistant points from two adjacent gait starting and stopping times were recorded as walking and resting points. This data segmentation procedure was inspired by similar approaches reported in [22, 23, 52]. Windows of different time lengths were chosen to correspond to the starting, stopping, walking and resting times thus obtained. To analyze the feasibility of a fully predictive intention recognition system, two kinds of data windows were extracted for classifier training and cross-validation. For fully predictive system, three types of windows were evaluated: [-1, 0], [-1.5, 0] and [-2, 0] second windows where no data after the movement initiation were used. Also, [-1, 1] and [-1.5, 0.5] second windows were extracted and validated where a brief portion of data after the occurrence of movement starting or stopping was used for feature extraction and classification. The numbers represent the starting time and ending time of the window corresponding to the event onset time, i.e. [-1,1] second window represents a two-second window starting one second before and ending one second after the ‘starting’ and ‘stopping’ of gait events. For the sake of balanced and unbiased classification, the length of ‘resting’ and ‘walking’ data samples were set as equal to that of the ‘starting’ and ‘stopping’ classes. It was made sure that there was no overlap between consecutive data samples belonging to different classes After that, two two-class classification problems were addressed: ’Rest vs. Start’ and ’Walk vs. Stop.’ This segmentation and windowing approach ensured no overlapping between data windows corresponding to different classes reducing the chance of data contamination.

### Feature extraction

For extracting distinguishing features from the non-stationary EEG data; the segmented data windows obtained in the previous subsection were further divided into 0.5-s long data windows with 50 milliseconds overlap. Thus, each one-second epoch was divided into 11 windows, 1.5 s windows were divided into 21 sub windows and 2 s windows yielded 31 sub windows. All three Hjorth parameters were computed for both alpha and beta band signals for the resulting sub windows. Thus, for every one-second data window, a total of 11 windows ×2 sub-bands ×3 parameters = 66 features were calculated per channel. Similarly, a total of 126 and 186 features were generated for 1.5 s and 2 s data windows. All the features corresponding to all the channels were then concatenated to form the final feature vector. Equations (1)-(6) summarizes the feature extraction, and the subsequent paragraph discusses feature vector formation. Equations (1) and (2) shows the activity parameters, Eqs. (3) and (4) defines the mobility parameters and Eqs. (5) and (6) describes the complexity parameters.

1 $$\begin{array}{*{20}l} A_{j,k,\alpha}^{i} = \sigma^{2}_{x^{i}_{j,k,\alpha}} \end{array} $$

2 $$\begin{array}{*{20}l} A_{j,k,\beta}^{i} = \sigma^{2}_{x^{i}_{j,k,\beta}} \end{array} $$

3 $$\begin{array}{*{20}l} M_{j,k,\alpha}^{i}=\frac{\frac{\sigma_{dx_{j,k,\alpha}^{i}}}{dt}}{\sigma_{x_{j,k,\alpha}^{i}}} \end{array} $$

4 $$\begin{array}{*{20}l} M_{j,k,\beta}^{i}=\frac{\frac{\sigma_{dx_{j,k,\beta}^{i}}}{dt}}{\sigma_{x_{j,k,\beta}^{i}}} \end{array} $$

5 $$\begin{array}{*{20}l} C^{i}_{j,k,\alpha}=\frac{\frac{\sigma_{d^{2}x^{i}_{j,k,\alpha}}}{dt}}{\frac{\sigma_{dx^{i}_{j,k,\alpha}}}{dt}}M^{i}_{j,k,\alpha} \end{array} $$

6 $$\begin{array}{*{20}l} C^{i}_{j,k,\beta}=\frac{\frac{\sigma_{d^{2}x^{i}_{j,k,\beta}}}{dt}}{\frac{\sigma_{dx^{i}_{j,k,\beta}}}{dt}}M^{i}_{j,k,\beta} \end{array} $$

Here *x* denotes the EEG signal. *A*, *M* and *C* denote the activity, mobility and complexity parameters, and *σ* denotes standard deviation of *x*. While *i*, *j* and *k*, denote the channel number, sample number, and window number respectively. All the features corresponding to a single sample from all the channels are concatenated to form the initial feature vector.

### Feature selection

As the number of features is too high compared to the number of samples, there is a high chance of having redundant and noisy features in the feature set. That is why a feature selection method is necessary to get rid of the redundant features. In this work, the absolute value of the standardized u-statistic of a two-sample unpaired Wilcoxon test [53], (also known as the Mann-Whitney test) was chosen to be the criterion to select distinctive and informative features. To further reduce the number of features, the average of the absolute values of the cross-correlation coefficient between the candidate feature and all previously selected features were calculated, and features that were highly correlated with the features already picked were less likely to be included in the output list. This procedure ensured the formation of a reduced and more distinctive set of features for successful classification. After feature selection, the number of selected features was reduced to 20 for all the subjects and classes.

### Classification

The ultimate goal of this study is to come up with a feasible methodology to apply in real-time BCI systems. For a proper real-time BCI system, the classifiers must have high sensitivity as well as high specificity to avoid accidents while using a prosthesis or orthosis system. With that in mind, in this work, we used an SVM classifier [54–56] with RBF kernel to solve two two-class supervised classification [57] problems. For each subject, one classifier was trained to classify between ‘rest’ and ‘start’ classes while another classifier was trained to classify between ‘walk’ and ‘stop’ classes. The performance of the classifiers was evaluated by ten-fold cross-validation. In each step of classification, one fold was used as the test set while all the other folds were used to train the classifier. Each fold was used only once as the test set, and finally, the performance metrics were average across all the folds.

## Results

For performance evaluation, the following metrics were calculated in this study: Accuracy, sensitivity, specificity. Accuracy shows the performance of the classifier in predicting start or stop and walk or rest classes. Sensitivity is the measure of the capability of the classifiers of correctly predicting the start or stop class, while specificity is the measure of the classifier performance in successfully predicting the walk or rest classes. The definition of these parameters are included in the following equations:

7 $$\begin{array}{*{20}l} Accuracy = \frac{TP+TN}{TP+TN+FP+FN} \end{array} $$

8 $$\begin{array}{*{20}l} Sensitivity=\frac{TP}{TP+FN} \end{array} $$

9 $$\begin{array}{*{20}l} Specificity=\frac{TN}{TN+FP} \end{array} $$

Here TP, TN, FP, FN denotes true positive, true negative, false positive and false negative detection where the ‘start’ and ‘stop’ windows belong to ‘positive’ class and ‘resting’ and ‘walking’ data windows belong to the ‘negative’ class. Another informative metric is FP/min. FP/ min is the ratio of the number of false detection of intention to start or stop and the number of rest or walk trials per minute. The resulting average performance parameters are summarized in this section.

**Fig. 4:**
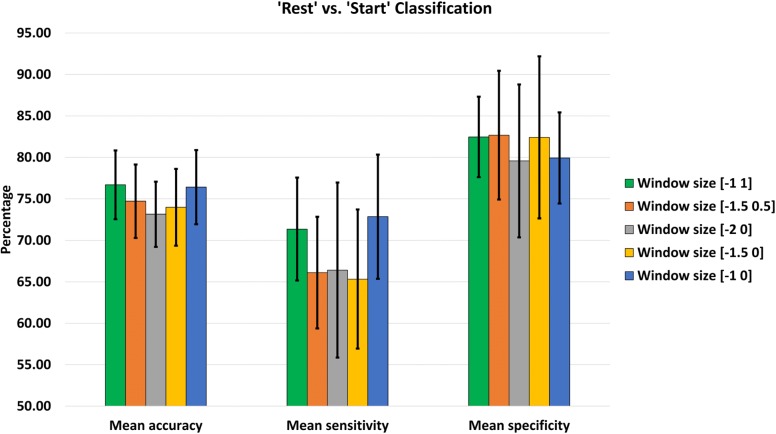
‘Rest’ vs. ‘Start’ classification performance with standard deviation using different data windows

**Fig. 5:**
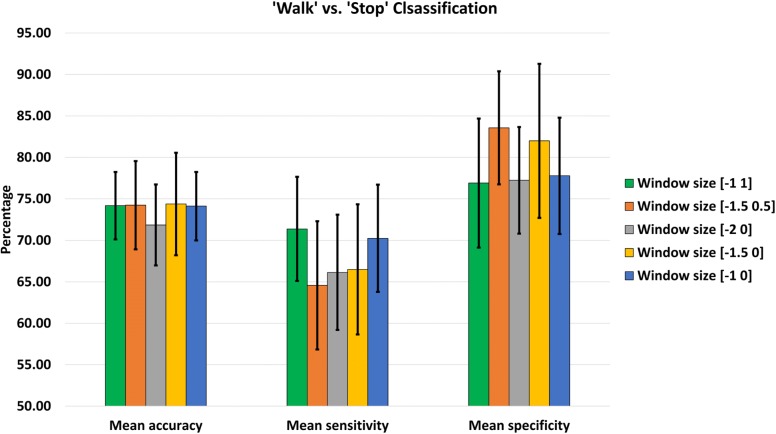
‘Walk’ vs. ‘Stop’ classification performance with standard deviation using different data windows

Tables 3 and 4 summarizes the accuracy, sensitivity, and specificity for the ‘Rest’ vs. ‘Start’ and ‘Walk’ vs. ‘Stop’ classification respectively for different data windows across all the subjects. Tables 5 and 6 show the average classification accuracy, sensitivity, and specificity across the subjects along with the standard deviations. In all of these tables, the first two columns represent the performance of the trans-event windows, while the latter three show the classification performance of the pre-event data windows only. The highest performance metrics obtained in both cases are highlighted in bold fonts. For the sake of understanding the results better, the results are also presented in Figs. 4 and 5.

**Table 3:** ‘Rest’ vs. ‘Start’ classification accuracy, sensitivity and specificity for all the subjects using different data windows

	Subjects	Data windows				
Accuracy(%)		[-1 1]	[-1.5 0.5]	[-2 0]	[-1.5 0]	[-1 0]
	S1	**80.63**	75.46	73.07	74.77	**80.30**
	S2	**73.76**	69.23	70.71	66.18	**75.19**
	S3	**83.35**	76.45	74.12	76.15	**82.67**
	S4	71.20	**71.82**	66.66	**72.16**	69.81
	S5	73.52	**75.81**	74.31	**75.41**	74.97
	S6	75.13	**78.25**	73.04	75.00	**77.63**
	S7	**75.22**	73.74	71.65	71.97	**73.45**
	A1	**75.88**	68.88	**73.17**	70.98	71.96
	A2	81.49	**82.80**	81.55	**83.21**	81.73
Sensitivity(%)	S1	**75.14**	63.00	58.71	58.90	**73.71**
	S2	68.30	**68.35**	49.29	59.56	**77.80**
	S3	**80.14**	78.90	69.57	80.95	**83.67**
	S4	**65.43**	58.05	55.00	**69.48**	66.24
	S5	60.05	**71.65**	**81.59**	72.20	66.48
	S6	**70.54**	64.46	**76.43**	53.93	68.21
	S7	**73.52**	66.04	63.85	59.62	**72.80**
	A1	**71.61**	56.96	**73.21**	64.11	63.39
	A2	**77.50**	67.50	70.00	69.17	**83.33**
Specificity(%)	S1	86.19	**87.48**	87.52	**90.29**	86.95
	S2	**79.23**	70.11	**92.14**	72.80	72.58
	S3	**86.29**	74.05	78.43	71.29	**81.43**
	S4	76.95	**85.57**	**77.86**	74.95	73.57
	S5	**86.76**	79.95	67.14	78.41	**83.30**
	S6	79.46	**92.14**	68.93	**95.89**	86.96
	S7	76.92	**81.26**	79.29	**84.12**	74.07
	A1	**80.36**	80.18	73.21	77.32	**79.64**
	A2	90.00	**93.33**	91.67	**96.67**	80.83

**Table 4:** ‘Walk’ vs. ‘Stop’ classification accuracy, sensitivity and specificity for all the subjects using different data windows

	Subjects	Data windows				
Accuracy(%)		[-1 1]	[-1.5 0.5]	[-2 0]	[-1.5 0]	[-1 0]
	S1	**74.21**	74.15	70.64	73.83	**75.24**
	S2	**75.80**	70.85	68.46	70.54	**75.44**
	S3	**73.43**	72.94	69.36	71.18	**75.46**
	S4	69.05	**71.26**	65.96	**71.86**	68.41
	S5	70.15	**70.51**	70.30	70.51	**70.92**
	S6	**71.88**	69.79	**72.42**	70.42	71.25
	S7	**72.30**	71.94	69.78	**70.86**	70.54
	A1	79.83	**83.74**	79.79	**85.65**	79.74
	A2	80.89	**82.86**	80.00	**84.52**	80.06
Sensitivity(%)	S1	60.57	**68.52**	65.29	67.71	**72.05**
	S2	**72.97**	65.05	52.42	67.36	**74.40**
	S3	**74.81**	71.86	66.00	70.33	**82.19**
	S4	**72.43**	64.57	60.81	65.95	**60.71**
	S5	**72.09**	54.56	**70.82**	69.78	64.67
	S6	**71.79**	53.57	67.68	56.25	**72.86**
	S7	**61.81**	57.36	**64.89**	51.65	64.01
	A1	**76.79**	72.32	69.82	**76.07**	69.64
	A2	**79.17**	73.33	**77.50**	73.33	71.67
Specificity(%)	S1	75.67	**88.33**	**80.10**	80.05	78.52
	S2	**84.67**	78.68	**76.65**	73.68	76.37
	S3	72.24	**72.29**	**74.24**	72.10	68.76
	S4	**70.86**	65.76	77.86	**78.05**	75.62
	S5	**70.11**	68.02	**85.93**	70.82	77.25
	S7	82.80	**86.43**	74.89	**90.00**	77.09
	S6	**76.96**	71.43	**85.36**	84.46	69.29
	A1	**88.93**	82.32	**94.64**	**94.64**	88.75
	A2	80.83	**82.50**	90.83	**94.17**	88.33

**Table 5:** Mean classification accuracy, sensitivity and specificity with standard deviation for ‘Rest’ vs. ‘Start’ classification

	Data windows				
	[-1 1]	[-1.5 0.5]	[-2 0]	[-1.5 0]	[-1 0]
Mean accuracy(%)	**76.69** ±**4.14**	74.72 ±4.42	73.14 ±3.92	73.98 ±4.62	**76.41** ±**4.47**
Mean sensitivity(%)	**71.36** ±**6.19**	66.10 ±6.73	66.41 ±10.54	65.32 ±8.38	**72.85** ±**7.48**
Mean specificity(%)	82.46 ±4.86	**82.67** ±**7.77**	79.58 ±9.22	**82.41** ±**9.77**	79.93 ±5.50

**Table 6:** Mean classification accuracy, sensitivity and specificity with standard deviation for ‘Walk’ vs. ‘Stop’ classification

	Data windows				
	[-1 1]	[-1.5 0.5]	[-2 0]	[-1.5 0]	[-1 0]
Mean accuracy(%)	74.17 ±4.05	**74.23** ±**5.31**	71.86 ±4.88	**74.38** ±**6.17**	74.12 ±4.12
Mean sensitivity(%)	**71.38** ±**4.14**	64.57 ±7.74	66.14 ±6.95	66.49 ±7.84	**70.24** ±**6.45**
Mean specificity(%)	76.90 ±7.77	**83.56** ±**6.82**	77.24 ±6.42	**82.00** ±**9.28**	77.78 ±7.01

From Table 3, it is evident that the highest accuracy for ‘Rest’ vs. ‘Start’ classification among the healthy subjects was achieved for S3 which was 83.35% with a sensitivity of 80.14% and a specificity of 86.29% using [-1 1] data window which consisted of EEG data both before and after event occurrence. However, a similar classification accuracy of 82.67% was achieved using a pre-movement [-1 0] data window with balanced sensitivity and specificity of 83.67% and 81.43% respectively. Among the amputated subjects, the highest accuracy was reached for A2, which was 83.21% with a sensitivity of 69.17% and a specificity of 96.67% using [-1.5 0] data window. The highlighted results in Table 3 suggests that for every subject, there were one or more pre-movement data windows, which were resulting in almost similar classification results compared to trans-movement data windows. Table 5 shows that [-1 1] window yielded slightly better accuracy than the [-1 0] window with an overall accuracy of 76.69±4.14*%*, which was the highest among all the data windows. In terms of sensitivity, the highest value was obtained from [-1 0] data window, and the highest specificity was achieved for [-1.5 0.5] window. Figure 4 shows the pictorial representation of the information mentioned above.

In case of ‘Walk’ vs. ‘Stop’ classification, the highest accuracy of 75.80% was obtained for S2 among the healthy subjects using the [-1 1] data window comprising both trans-event data. The corresponding sensitivity and specificity values were 72.97% and 84.67%, respectively. Almost equal accuracy, sensitivity, and specificity values of 75.44%, 74.40%, and 76.37% were achieved for the same subject using only the pre-event [-1 0] data window. Among the amputated subjects, the best results were obtained for pre-event [-1.5 0] window for subject A1. The classification model achieved an accuracy of 85.65%, a sensitivity of 76.07% and a specificity of 94.64%. A slightly decreased classification accuracy was achieved for this subject using trans-event window [-1.5 0.5]. The resulting accuracy, sensitivity, and specificity values were 83.74%, 72.32%, and 82.32%. Highlighted results in Table 4 suggest that the best performing trans-event data window had a similar or better performing pre-event data window for all the subjects. From Table 6, it can be seen that the highest mean accuracy for ‘Walk’ vs. ‘Stop’ classification was achieved using [-1.5 0] data window, which was 74.38±6.17*%*. The highest sensitivity was achieved using the [-1 1] window, and the highest average specificity was achieved using [-1.5 0] window.

A two-sample t-test with Bonferroni-Holm correction [58] was also performed between the classification results obtained from the trans-event data windows and those obtained from only the pre-event data windows. The hypotheses of the test were:

*H0: Mean classification performance of trans-event data windows is equal to that of pre-event data windows.*

*H1: Mean classification performance of trans-event data windows is greater than that of pre-event data windows.*

For ‘Rest’ vs. ‘Start’ classification, the t-test result yielded that the mean accuracy obtained by only the [-1 1] data window was significantly better than those of [-2 0] and [-1.5 0] data windows at *p*<0.05 significance level. All the other pairs could not reject the null hypothesis. This result indicates to the idea that the pre-movement window of [-1 0] seconds can lead to statistically similar gait start intention detection accuracy compared to the trans-event data windows namely [-1 1] and [-1.5 0.5] data windows. On the other hand, for ‘Walk’ vs. ‘Stop’ classification, the t-test result showed that the mean detection accuracy obtained by using the [-1 1] and [-1.5 0.5] windows are only significantly better than that of the pre-event [-2 0] data window. The other pre-event data windows have statistically similar detection accuracy compared to the trans-event data windows. The t-test results are summarized in Table 7. The results of the statistical analysis show that it is possible to obtain similar gait start or stop intention detection by using either the combination of data windows before and after the event or only the data windows before the events at *p*<0.05 significance level and hence support the hypothesis of the study.

**Table 7:** Result of t-test with Bonferroni-Holm correction

’Rest’ vs. ’Start’	Data Window	[-2 0]	[-1.5 0]	[-1 0]
	[-1 1]	H1	H1	H0
		*p*=0.0011	*p*=0.0080	*p*=0.3990
	[-1.5 0.5]	H0	H0	H0
		*p*=0.0744	*p*=0.2474	*p*=0.9398
’Walk’ vs. ’Stop’	[-1 1]	H1	H0	H0
		*p*=0.0067	*p*=0.5901	*p*=0.4762
	[-1.5 0.5]	H1	H0	H0
		*p*=0.0058	*p*=0.5658	*p*=0.4514

Overall average true positive rate achieved in the study was 72.06±8.27*%*. For all the participants, the total number of rest or walk trials was 1564, and the total recording time was 227.4 minutes. This calculation amounts to 6.88 rest or walk trials per minute. The resulting FPR was approximately 1.45/min on an average.

## Discussion

This study aims to analyze the performance of the pre-movement EEG signals in predicting human intention for gait initiation or termination. Also, this study hypothesized that it is possible to obtain similar intention detection performance, whether post-event EEG data were used for classification or not. The ability to predict gait intention is a very significant feature to have to design and implement a prosthetic or rehabilitation system with potential real-life application. Because the earlier the intention state of any human subject can be recognized, the earlier the prosthetic system parameters can be adapted and prepared according to the subject‘s needs.

In this paper, ICA was used along with other algorithms to clean the EEG data. However, the computational complexity involved in EEG source analysis by combining ICA and blind source localization is quite significant. That is why most of the ICA based EEG analysis tools offers offline processing. However, recently there has been an introduction and demonstration of Real-time EEG source mapping toolboxes, e.g. REST [59], Online Recursive ICA (ORICA) [60] which use recursive independent component analysis [61] to estimate a solution to the source separation problem in near real-time, allowing low latency access to source information. Thus, the recent technologies have shown promise to make possible innovations in experimental designs for a lot of BCI systems. In addition to that, traditional spatial filtering techniques including Laplacian filtering and common average referencing are also available as alternative [22, 23, 52]. These filters aim to minimize the contribution of the rest of the EEG electrodes to each channel thus better isolating the information from each of the electrodes. Such spatial filtering technologies can be useful alternatives until an enhanced and robust online ICA algorithm is developed for real-time application.

Moreover, the window equidistant between the ‘start’ and ‘stop’ windows were taken as ‘walking’ and ‘resting’ windows in this study. The lengths of ‘walk’ and ‘rest’ periods were bigger than those of ‘start’ and ‘stop’ periods by a great margin. Usage of the whole chunk of data corresponding to ‘walk’ and ‘rest’ period for classification would have led to imbalanced classes making the classification result highly biased. To ensure the formation of balanced classes, ‘walk’, ‘rest’, ‘starting’ and ‘stopping’ data windows were selected so that they were of the same length. Additionally, we wanted to measure the separability of intention of ‘starting’ and ‘stopping’ data from steady state ‘walking’ and ‘resting’ data in this study. Due to subjective variability and data non-stationarity, the initial changes corresponding to gait starting and stopping might cause interference to the walking and stopping data if those windows were chosen immediately before the gait windows. Such condition would have significantly affected the outcome of the study. Therefore, we ensured that the ‘walking’ and ‘resting’ data were not corrupted by neural waves caused by intention of gait by choosing data windows far from the ‘start’ and ‘stop’ data windows. In an online scenario the data would be accessed asynchronously and each data window would be assessed independently which would be included in the future work related to the study.

The two-sample t-test with Bonferroni-Holm correction suggests that for both ‘Rest’ vs. ‘Start’ and ‘walk’ vs. ‘Stop’ classification, it was possible to yield similar classification performances using either trans-event or only pre-event EEG signals. This, in turns, shows that the addition of post-event data windows does not always add much statistical value to the intention detection methodology. This outcome is a very significant one because being able to predict human gait intention using the data before the movement only gives much more preparation time for prosthetic system preparation and proper operation which is a key to successful prosthesis and neuro-rehabilitation. It is to be noted that significance in t-test does not guarantee a similar performance in real life, rather an online study should be operated to evaluate the validity of the outcome of this offline study. However, t-test is a widely used inferential statistic used to calculate and evaluate the probability of difference between two sets of data. In this study, the t-test was used to determine whether, statistically speaking, the distribution of accuracies obtained from the “pre-movement” data windows and “trans-movement” data windows are statistically different or not. The use of t-test is significant in the scope of this study to quantify the statistical significance of difference in performance obtained using the two types of intention detection models. For instance, even if the accuracy resulting from either of cases is greater than the other by a very small margin it can simply arise from modeling bias and thus no conclusion about superiority or similarity of performance can be drawn from mere visual inspection. Therefore, there must be some statistical measure to evaluate the significance of the obtained results and t-test serves that purpose in this study. Probabilistically, the results of the t-tests at 5% significance level implies that there is at least 95% probability that the two sets of results in contention come from different distributions thus have statistical difference from each other.

In this study, the instantaneous time-frequency information was used for gait intention detection. Now, neurological studies regarding voluntary human movement suggest that gait-related changes in human brain waves may start 1.5-2 s before the movement and may sustain till 2 s after the movement. The initial change in neural waves originates from motor preparation in the motor cortex which is strengthened by sensory feedback immediately after the movement. Due to the subject dependent variability in motor preparation and execution, the time of initial changes related to gait preparation may vary. Nevertheless, such physiological changes are very strongly present close to the moment of movement initiation and termination which diminish with time. Therefore, the intention of gait initiation and termination is expected to be detected more successfully from the data windows close to the time of event. In our study, the EEG information originating from data windows closest to the event, namely the [-1,0] and [-1,1] second windows resulted in the best results. The results comply with the neurological facts established by previous neurological studies. The overall statistics show that for both ’walk’ vs. ’stop’ and ’rest’ vs. ’start’ classification problems, the [-1,1] and [-1,0] windows achieved a comparatively more balanced performance in terms of sensitivity and specificity. This is a very significant outcome as this suggests that these two windows contained comparatively more distinguishable features. This result further shows the potential of these data windows in real time application. The other data windows, namely [-2,0], [-1.5,0.5] and [-1.5,0] included more data from before the event of gait which might be responsible for corrupting the classification models by introducing randomness and thus skewing the obtained classification hyper-plane.

For all the detection models, the average specificity was higher than the average sensitivity values. These values mean that the system was able to detect the ‘walking’ and ‘resting’ windows much more successfully than the ‘start’ and ‘stop’ windows. The highest sensitivity was obtained from [-1 0] data window for ‘Rest’ vs. ‘Start’ and the highest sensitivity for ‘Walk’ vs. ‘Stop’ was reported from [-1 1] window. However, achieving only higher sensitivity does not ensure safe operation of a prosthetic system. Instead, a low specificity results in higher False Positive Rate (FPR), which can be very hazardous to the person using the prosthetic system. That is why while training the classification models, a higher cost was assigned to the wrong detection of intention. This precautionary action was taken to prevent a high false positive detection rate which can lead to high risk for a user of prosthetic system. The adjusted cost-sensitive SVM model can be presented as follows [62]:

10 $$\begin{array}{*{20}l} min \frac{1}{2}\|{w}\|^{2} + C^{+}\sum_{i \epsilon{class1}}{e(y_{i},p_{i}))}+C^{-} \sum_{i \epsilon {class2}}{e(y_{i},p_{i}))} \end{array} $$

11 $$\begin{array}{*{20}l} y_{i} (w^{T} \phi (x_{i}) + b) >= 1-\zeta_{i} \end{array} $$

Here, *w* nad *b* denotes the normal vector and bias of the separator hyper-plane. *ζ*_*i*_ denotes the slack variables, *x* and *y* represent the training data and corresponding classes, *p* denotes the predicted class of the training sample, *ϕ*(*x*) denotes the kernel function, class 1 and class 2 denote the two classes of the binary classification problem, *C*^+^ and *C*^−^ denote the misclassification rates associated with class 1 and class 2, and *e*(*y*_*i*_,*p*_*i*_) denote the error function. By setting a higher value to *C*^+^ for the ‘start’ and ‘stop’ classes compared to *C*^−^ for the ‘rest’ and ‘walk’ classes, the misclassification of ‘start’ and ‘stop’ was penalized more in the training process.

On top of that, the subjective heterogeneity in results might arise from the innate randomness and non-stationary nature involved in the gait related EEG data. The huge disparity between sensitivity and specificity in some cases originated from the extra cost put on false positive detection which made the intention detection criterion much stricter. This also suggests that in those cases the features corresponding to the ’start’ and ’stop’ classes did not hold enough distinguishable information to separate them from the ’rest’ and ’walk’ classes and thus could lead to erroneous detection in the daunting task of online gait intention detection. This resulted in slightly random labels for the testing samples leading to higher disparities in specificity and sensitivity values. Therefore, the average sensitivity is slightly lower than the average specificity. This limitation can be overcome by advanced machine learning techniques like ensemble learning or majority voting. For both the classification problems, the [-1 0] data window yielded the most balanced average sensitivity and specificity values, which shows the potential of this data window in real time application.

The average classification performance obtained for the amputated subjects were higher than those of the healthy subjects. Although the number of amputated subjects was low, the classification performances were consistently higher than the average performance for the healthy subjects. This occurrence might be due to the fact that, as the amputated persons were asked to start and stop their gait cycle on their prosthetic leg, it required more effort and concentration on their part. That is why it might result in easier detection of the intention of gait initiation or termination. Moreover, for the amputees, the best window for intention detection was found to be the [-1.5 0] window compared to the [-1 0] window for the healthy subjects. That points to the possibility that those amputated subjects appear to prepare for the upcoming change in gait state slightly earlier than their non-amputated counterparts. These are interesting questions which can be looked further into for insight about gait preparation and execution of the amputees for a better experimental design.

Neurological studies regarding voluntary human movement suggest that gait-related changes in human brain waves may start 1.5-2 s before the movement and may sustain till 2 s after the movement. The initial change in neural waves originates from motor preparation in the motor cortex which is strengthened by sensory feedback immediately after the movement. Due to the subject dependent variability in motor preparation, the time of initial gait related changes may vary. Nevertheless, such physiological changes are very strongly present close to the moment of movement initiation and termination which diminish with time. Therefore, the intention of gait initiation and termination is expected to be detected more successfully from the data windows close to the time of event.

In this study, two classifiers were trained to predict the intention to both start and stop. The two classifiers were termed as: ‘Walk’ vs ‘Stop’ and ‘Rest’ vs ‘Start’. The modeling of two separate classifiers opposed to a single binary classifier (‘Walk’ vs ‘Rest’) provides additional insights into the problem at hand. The neural changes related to intention of gait transition starts to appear while the person is still in the previous state of gait. For example, if a person is intending to start walking just before starting to walk, the EEG features will appear while he is still standing. Prediction of gait intention requires successful identification of the neural changes in those small windows. If only one model is trained to classify ‘Walk’ vs ‘Rest’ classes, there is a very high possibility that the pre-movement neural changes related to intention of gait transition would be misclassified. Moreover, that would turn the classification problem to steady state gait stage identification problem instead of the current intention prediction problem. In summary, modeling of two separate classification models enables a BCI system to have a higher probability to predict intention to both start and stop of gait compared to initial steady state walking or resting period.

Although there are very few studies with the same experimental design, a similar offline classification was reported in [22] with a TPR of 54.8% and 2.66 FP/min. Another study reported in [23] achieved an accuracy of 72.91% with 71.81 ±11.48% true positive rate and 4.56 ±1.84 FP/ min for start detection and accuracy, TPR and FP/min of 80.65 ±11.49%, 57.38 ±12.03% and 2.10 ±1.20 for stop detection. In a more recent study [52], the accuracy, sensitivity and FP/min were reported as 78.61 ±11.20%, 76.90 ±11.75% and 3.52 ±1.82 for start detection and 84.36 ±10.19%, 68.68 ±14.69% and 2.06 ±1.12 for stop detection. It is to be noted though that these studies used a very large detection window of 4- s containing data from two seconds before the starting and stopping event to two seconds after the occurrence of the event. The results suggest that the proposed method resulted in better or competitive accuracy, sensitivity with an enhanced specificity for start detection using a smaller pre-movement data window. For stop detection, the existing studies showed better accuracy, however the true positive rates were inferior to the ones achieved in this study. Thus the proposed study provides a balanced start and stop detection methodology without accessing the post event data windows. This enhanced accuracy with higher TPR and lower FP/ min show the prospect of the proposed method in successfully classifying gait starting or stopping intention vs. steady state walking or resting trials.

## Conclusion

This paper proposes a wavelet transform-based methodology using Hjorth parameters as features for predicting human intention for gait starting and stopping. A combination of ASR and ICA was carried out to clean the data off any non-brain artifacts or noises. As the results suggest, it was possible to generate statistically similar intention detection performance using only the pre-movement time windows. As a result, the obtained results show a promising ability to predict movement intention, even if the intentions are relatively sudden. The proposed methodology can be a good starting point for future studies to implement a real-time BMI system for assistive devices. However, the study simply evaluates how separable the ‘walking’ or ‘resting’ periods’ data windows are from ‘start’ or ‘stop’ walking data windows using the proposed methodology. Due to the extremely noisy and non-stationary nature of EEG signals accompanied by the subjective variability of gait preparation, the uncertainty involved in solving such detection problem is very high. This causes the classification performance to degrade significantly. For safe and robust control, asynchronous intention detection schemes need to be carried out and the performances need to be evaluated in terms of critical parameters necessary for real-time BCI application. Advanced signal processing techniques in addition to enhanced machine learning methodologies like threshold regulation, combination of decisions from multiple consecutive windows, neural networks can be applied to further increase the classification accuracy. Future works can be done in the scope of decreasing the false positive rates, increasing the accuracy, sensitivity, and specificity of the system, asynchronous prediction of movement intention and application of an enhanced methodology in real-time studies.

## Data Availability

The datasets generated during and/or analyzed during the current study are available from the corresponding author on reasonable request.

## Abbreviations

EEG: Electroencephalography
BCI: Brain computer interface
SVM: Support vector machine
TPR: True positive rate
EMG: Electromyography
BMI: Brain-machine interfaces
ERP: Event-related potentials
MRCP: Movement-related cortical potential
ERD/ERS: Event-related synchronization/ desynchronization
BP: Bereitschafts potential
FFT: Fast fourier transform
STFT: Short time fourier transform
IRB: Institutional review board
TA: Tibialis anterior
IMU: Inertial measurement unit
ASR- Artifact subspace reconstruction; ICA: Independent component analysis
SD: Standard deviation
AMICA: Adaptive mixture independent component analysis
FP: False positive
FPR: False positive rate
